# Real-world objects are more memorable than photographs of objects

**DOI:** 10.3389/fnhum.2014.00837

**Published:** 2014-10-20

**Authors:** Jacqueline C. Snow, Rafal M. Skiba, Taylor L. Coleman, Marian E. Berryhill

**Affiliations:** Cognitive and Brain Sciences Group, Department of Psychology, University of NevadaReno, NV, USA

**Keywords:** real-world, real objects, pictures, memory, recall, recognition memory

## Abstract

Research studies in psychology typically use two-dimensional (2D) images of objects as proxies for real-world three-dimensional (3D) stimuli. There are, however, a number of important differences between real objects and images that could influence cognition and behavior. Although human memory has been studied extensively, only a handful of studies have used real objects in the context of memory and virtually none have directly compared memory for real objects vs. their 2D counterparts. Here we examined whether or not episodic memory is influenced by the format in which objects are displayed. We conducted two experiments asking participants to freely recall, and to recognize, a set of 44 common household objects. Critically, the exemplars were displayed to observers in one of three viewing conditions: real-world objects, colored photographs, or black and white line drawings. Stimuli were closely matched across conditions for size, orientation, and illumination. Surprisingly, recall and recognition performance was significantly better for real objects compared to colored photographs or line drawings (for which memory performance was equivalent). We replicated this pattern in a second experiment comparing memory for real objects vs. color photos, when the stimuli were matched for viewing angle across conditions. Again, recall and recognition performance was significantly better for the real objects than matched color photos of the same items. Taken together, our data suggest that real objects are more memorable than pictorial stimuli. Our results highlight the importance of studying real-world object cognition and raise the potential for applied use in developing effective strategies for education, marketing, and further research on object-related cognition.

## Introduction

Our current scientific knowledge in areas such as human visual perception, attention, and memory, is founded almost exclusively on experiments that rely upon 2D image presentations. However, the human visuomotor system has largely evolved to perceive and interact with real objects and environments, not images (Gibson, 1979; Norman, 2002). Despite many fundamental differences between real objects and images, there has been very little investigation of whether real objects have a unique influence on cognition and action compared with pictorial displays. In the domain of human memory, studies have used real world objects (e.g., Dirks and Neisser, 1977; Mandler et al., 1977; Pezdek et al., 1986; Droll and Eckstein, 2009), but to our knowledge none have specifically examined whether memory performance is superior for real objects vs. matched image displays. In other words, the underlying and unexplored assumption is that representations of real objects are remembered equivalently to real objects. However, real objects may have a memory advantage that is important to consider, both for empirical reasons, and because of the potential benefits in other domains—such as education and marketing.

Real objects differ from pictures in a number of important respects, several of which could influence memory. First, real objects (when viewed with two eyes) possess additional cues to 3D shape than 2D pictures. When we look at the world with two eyes, each receives information about objects from a slightly different horizontal viewpoint—the geometrical discrepancy between which is known as binocular disparity (Harris and Wilcox, 2009; Blake and Wilson, 2011). The brain is able to resolve the discrepancy in these two images to produce a unitary sense of depth (Blake and Wilson, 2011). Conversely, when we view a static 2D picture of an object, no additional information about the depth structure of the object is available, and consequently we experience the stimulus as being “flat.” Further, 2D images present the visual system with inherent cue conflicts; monocular cues to 3D shape, such as surface texture, specular highlights, and linear perspective, indicate that the stimulus has depth, whereas binocular cues indicate that the stimulus is flat (Vishwanath and Kowler, 2004). Stimuli that lack stereo cues can profoundly disrupt object recognition in brain damaged patients. For example, visual agnosia patients are better at recognizing real objects than 2D pictures (Riddoch and Humphreys, 1987; Young and Ellis, 1989; Servos et al., 1993; Humphrey et al., 1994; Chainay and Humphreys, 2001; Hiraoka et al., 2009)—an effect that has been argued to be due to the additional depth cues inherent to real exemplars (Servos et al., 1993; Chainay and Humphreys, 2001). It is possible therefore, that additional information about the geometrical structure of real objects could facilitate memory compared with 2D image displays.

Second, unlike pictures, real objects afford action such as grasping and manipulation. In terms of neural responsivity, viewing real objects and images of objects activates similar networks, particularly the lateral occipital complex along the lateral and ventral convexity of occipito-temporal cortex (Snow et al., 2011). However, because real objects afford action, they can have a unique effect on neural responses. For example, when viewing object images, stimulus repetition leads to a characteristic reduction in fMRI responses—an effect known as fMR-adaptation or repetition suppression (RS) (Grill-Spector and Malach, 2001; Malach, 2012). Yet, recent research has highlighted important differences in RS as a function of the type of stimulus presented; RS for real objects is weak, if not absent, when observers view *real* objects compared to matched 2D photographs of the same items (Snow et al., 2011). Further, some brain areas, such as superior parieto-occipital cortex, respond differently depending on whether or not a graspable object is within reach of the dominant hand, regardless of whether or not a grasp is planned or executed (Gallivan et al., 2009, 2011). It is reasonable to suspect therefore that real-world, graspable objects are stored, represented, and/or processed differently than images of objects (Snow et al., 2011). Moreover, the potential for a motor interaction with real objects could strengthen or enhance the associated memory trace by automatically enhancing depth of processing at encoding (Craik and Tulving, 1975).

Third, real objects have an actual or veridical size, distance, and location relative to the observer, whereas images do not, and these cues to object identity could facilitate memory. Images—although often described as having a “real world size”—have only an *expected* size based on our experience with other similar exemplars in the natural environment (Konkle and Oliva, 2011, 2012b; Brady et al., 2013). As a consequence, when viewing images there is often a striking discrepancy between retinal size and real-world size, relative to the distance of the image from the observer. For some types of displays, such as scenes that possess background contextual information, we can make inferences about the relative size of objects depicted in the image (i.e., “the cow is smaller than the tree, but larger than the sheep”) yet the stimuli lack an *actual* size that would be relevant for motor planning. In most behavioral and neuroimaging studies, object stimuli are presented in isolation without background context making real world size even less apparent (i.e., is that a toy-sized object or is it real-life sized?) (e.g., Konen and Kastner, 2008). When background information *is* provided, retinotopic regions in the dorsal stream do track perceived distance (Berryhill and Olson, 2009). Thus, knowing the size, distance and location of a stimulus has consequences for the way in which it is perceived and this shapes future neural processing for cognition, action, and memory.

Given the fundamental differences between real objects and image displays outlined above, we wondered whether or not observers would show enhanced memory for everyday objects displayed as real exemplars vs. pictures. In the current study, we examined episodic memory performance by testing free recall and recognition for common household objects encoded under different viewing conditions. In Experiment 1 healthy college-aged students studied objects that were either viewed in the form of real world exemplars, high-resolution color photographs, or black and white line drawings. The line-drawing condition was included to determine the extent to which color and monocular cues to 3D shape (such as shading, and surface texture—all of which were present in the color photograph condition) bolster memory performance. Importantly, the stimuli in our study were closely matched for size, illumination, and orientation across the different viewing conditions. In Experiment 2, we controlled for the viewing angle at which the real objects and matched colored photographs were presented. Stimuli in all experiments were presented within arm's reach and viewed in their real world size. We predicted that if there were a real object benefit it would be reflected in significantly better memory performance. We included two measures of episodic memory, free recall and recognition, to assess more comprehensively the nature of any performance differences.

## Materials and methods

### Participants

One seventy two second-year psychology students at the University of Nevada, Reno participated in the studies, in exchange for course extra-credit (80 subjects Experiment 1; 92 subjects Experiment 2). All subjects gave informed assent or consent and the experimental protocols were approved by the University of Nevada, Reno Social, Behavioral, and Educational Institutional Review Board. There were no potential conflicts of interest as there were no commercial or financial benefits for any party.

### Materials

**Figure 1:**
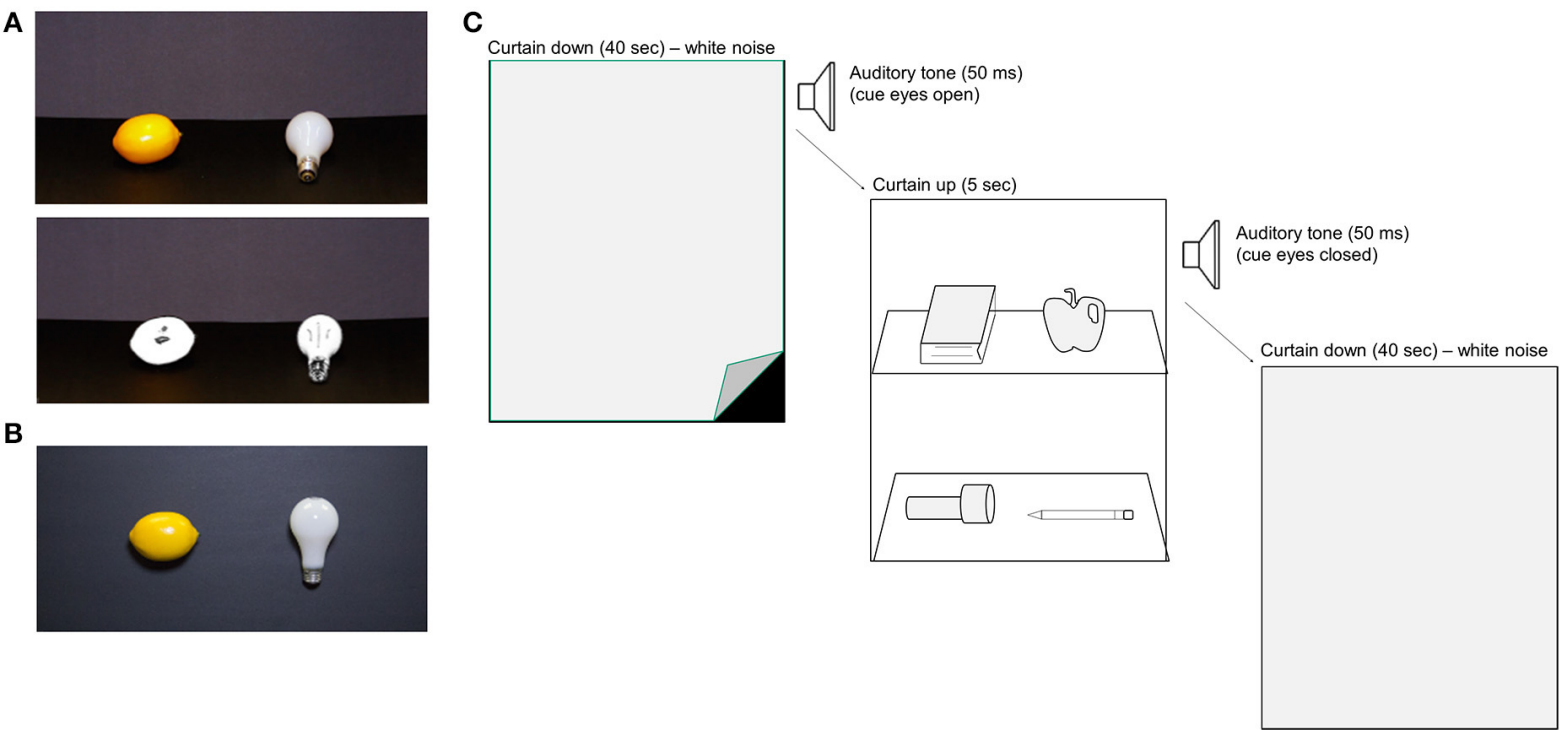
**Stimulus setup and trial sequence for Experiments 1 and 2. (A)** Two common household objects were presented on each shelf of the presentation box, for a total of four stimulus items per display. Observers stood within reaching distance of the display items. In Experiment 1, the stimuli were presented to observers in one of three Viewing Conditions: real objects (not shown), color photos (upper panel), or black and white line drawings (lower panel). The shelves were tilted ~30° toward the subject to facilitate recognition. **(B)** In Experiment 2, participants viewed either real objects or color photographs of the same objects, this time with the shelves positioned vertically to match stimulus viewing angle (example color photograph shown). **(C)** Trial sequence in the Study Phase. Observers viewed each stimulus display for 5 s, followed by a 40 s ITI. An auditory tone signaled subjects to close their eyes during the ITI, and a second tone signaled the beginning of the next trial. White noise was played during the ITI. A curtain was used to mask the stimuli from subjects view at the end of each trial and the display box was turned to face the experimenters during stimulus changeover. The Study Phase comprised of 11 stimulus trials (four items per display), yielding a total of 44 different objects.

The stimuli in each Experiment consisted of 44 common household objects and high-resolution photographs of the same items (Figure 1). The photographs were reproduced in color, and as black and white line-drawings. In Experiment 1 we compared memory performance for objects in three different Viewing Conditions: real objects, color photographs, and line-drawings. In Experiment 2 we compared memory for real objects vs. color photographs. Photograph (and line drawing) stimuli were matched to the real objects in terms of size, and orientation using the methods described below. Line drawings of each stimulus were created using Adobe Photoshop to remove all color and most surface texture cues by isolating the object in the image, using the Sketch-Photocopy filter, and raising contrast values in the image. Stimuli in each Viewing Condition were presented to observers within a custom display box; the boxes were constructed from black foam-core, and a gray curtain (that covered the entire display) was attached to the top of the box. Each real object was attached to a removable black foam-core shelf that could be quickly inserted into position within the display box. Each shelf held two objects, and each box held two shelves (upper/lower), yielding a total of four stimuli per trial. We used a Canon Rebel T2i DSLR camera with constant F-stop and shutter speed to photograph the real objects, separately for each shelf, thereby matching for stimulus orientation between the real object and photograph conditions. Image size was adjusted using Adobe Photoshop, and the resulting photograph stimuli were printed to match the real objects in size. In Experiment 1, the real object shelves were positioned at a 45° angle within the display box, and photographs of the shelves were taken at the same display angle (Figure 1A). In Experiment 2, the object stimuli were attached to shelves that were positioned in a *vertical* orientation, to match the real object and photograph stimuli for viewing angle (Figure 1B). Photograph stimuli were printed on HP Satin Q8923 paper and attached to shelves of identical size to the real object displays using double-sided tape. The timing of events in both experiments was controlled using Matlab (Mathworks, USA) and Psychtoolbox software packages.

### Procedure

We used a between-subjects design to compare memory performance in each Viewing Condition. The procedure was identical in Experiments 1 and 2 (Figure 1C). Subjects were assigned randomly to one Viewing Condition (Experiment 1: real objects: *n* = 26, color photographs: *n* = 27, line-drawings: *n* = 26; Experiment 2: real objects: *n* = 46, color photographs: *n* = 46), and each group was tested in a separate room. Subjects were not informed of the Condition to which they had been assigned, or to the fact that there were different Viewing Conditions in the experiment. Identical display boxes were situated in each testing room, and two experimenters were present in each room to run the study. During the Study Phase, participants were instructed to stand facing the display box at a distance of ~56 cm, with the stimuli within arm's reach. A 50 ms auditory tone (1000 Hz) signaled trial onset, at which time the experimenters lifted the curtain to reveal the stimuli. The shelves were displayed for 5 s, after which a second 50 ms auditory tone (500 Hz) signaled subjects to close their eyes during the 40 s ITI. At the onset of the second tone, the experimenters dropped the curtain, turned the display box around (facing away from the subjects), and prepared the display box for the upcoming trial. White noise was played throughout the ITI to mask extraneous noise during stimulus changeovers. Stimuli were presented in the same position, shelf, and in the same sequential order, in each Viewing Condition. Eleven trials were presented, for a total study set of forty-four objects.

In the subsequent Test Phase, participants were instructed to remember as many items as they could from the Study Phase. To control for recency effects (Bjork and Whitten, 1974) participants were first given a *semantic memory task* in which they were given 1 min to write down as many US states as they could. Next, in the *free-recall task*, participants were given 5 min to write the names of all objects they could remember from the Study Phase. All participants had finished the recall task to the best of their ability before the end of the 5-min time limit. Finally, subjects were given 10 min to complete a *recognition task* in which they were given a printed list of 88 object names, 44 of which were objects presented in the Study Phase, and the remaining 44 were distractor objects. The subjects' task was to judge whether or not each item had been presented in the Study Phase (True/False).

Participant responses were scored as either correct or incorrect (score /44 for the free recall task, and /88 for the recognition task). The mean number of falsely recalled items was also calculated for both experiments. Importantly, to control for any difficulty observers may have had in recognizing the stimuli (particularly the line-drawing condition), we conducted an item analysis. The number of times each stimulus was correctly recalled or identified was calculated separately for each Viewing Condition and Task. Next, mean recall and recognition performance was calculated across all items in the set for each Viewing Condition and Task, and objects for which memory performance fell below 3 standard deviations from the mean were eliminated from further analyses. Using this method, no items were removed from the recall task in either Experiment. For recognition, in Experiment 1 one distractor item (“Wrench”) was removed due to the high number of false positive responses, and in Experiment 2 two study items were removed from further analysis (“Trowel” and “Hat”) due to the high number of misses—possibly due to our subjects' vernacular for these terms, given that their free recall for the same items (using terms such as “Garden Shovel” and “Beanie”) was relatively high (see Tables 1, 2). Recognition performance in each Experiment was analyzed according to percent (%) correct, and using a signal detection (SD) analysis to disentangle sensitivity to the stimuli from possible effects of response bias (Green and Swets, 1974).

(1) $${\text{d}}^{\prime }=\text{z}(\text{H})\;-\text{ z}(\text{F})$$

**Table 1:** **Recall data for each stimulus item in Experiment 1**.

**Item**	**% Correct**			**Quartile**		
	**Real object**	**Color photo**	**Line drawing**	**Real object**	**Color photo**	**Line drawing**
Apple	80.77	66.67	42.31	4	4	3
Ruler	76.92	48.15	53.85	4	3	4
Soap	76.92	25.93	7.69	4	2	1
Paint roller	73.08	55.56	53.85	4	4	4
Flashlight	69.23	44.44	42.31	4	3	3
Lemon	69.23	48.15	30.77	4	3	2
Rubber duck	69.23	74.07	30.77	4	4	2
Dice	65.38	37.04	46.15	4	3	3
Paintbrush	65.38	55.56	53.85	4	4	4
Book	61.54	66.67	50.00	3	4	4
Corkscrew	61.54	11.11	19.23	3	1	1
Oven mitt	61.54	62.96	50.00	3	4	4
Pencil	61.54	74.07	73.08	3	4	4
Hairbrush	57.69	7.41	57.69	3	1	4
Calculator	53.85	14.81	23.08	3	1	1
Comb	53.85	25.93	53.85	3	2	4
Cork	53.85	37.04	19.23	3	3	1
Glove	53.85	29.63	30.77	3	2	2
Mug	53.85	29.63	19.23	3	2	1
Screwdriver	53.85	29.63	50.00	3	2	4
Toothbrush	53.85	29.63	46.15	3	2	3
Hat	50.00	51.85	53.85	2	4	4
Hole punch	50.00	25.93	30.77	2	2	2
Plate	50.00	44.44	53.85	2	3	4
Spoon	50.00	18.52	38.46	2	1	3
Highlighter	42.31	81.48	30.77	2	4	2
Small shovel (trowel)	42.31	25.93	30.77	2	2	2
Tennis ball	42.31	33.33	46.15	2	3	3
Glasses	38.46	62.96	42.31	2	4	3
Pizza cutter	38.46	11.11	11.54	2	1	1
Pliers	38.46	22.22	38.46	2	1	3
Rubber spatula	38.46	37.04	26.92	2	3	2
Bottle	34.62	48.15	30.77	1	3	2
Funnel	34.62	14.81	23.08	1	1	1
Light bulb	34.62	44.44	26.92	1	3	2
Shell	34.62	29.63	19.23	1	2	1
Tape dispenser	34.62	25.93	34.62	1	2	3
Ladle	30.77	44.44	15.38	1	3	1
Magnifying glass	30.77	11.11	7.69	1	1	1
Sponge	30.77	18.52	23.08	1	1	1
Salt shaker	26.92	33.33	26.92	1	3	2
Nail file	23.08	14.81	11.54	1	1	1
Scissors	19.23	22.22	23.08	1	1	1
Bell	15.38	14.81	7.69	1	1	1
Grand mean	49.04	36.62	34.27	-	-	-

Percent (%) correct recall and recall quartile are displayed separately for each Item and Viewing Condition. Data are ranked according to % correct recall for real object displays. Note that items with a higher quartile ranking (i.e., 4th) are recalled more frequently than those in lower quartiles (i.e., 1st).

**Table 2:** **Recall data for each stimulus item in Experiment 2**.

**Item**	**% Correct**		**Quartile**	
	**Real object**	**Color photo**	**Real object**	**Color photo**
Apple	89.13	62.50	4	4
Glove	80.43	68.75	4	4
Pear	80.43	72.92	4	4
Pencil	78.26	81.25	4	4
Ruler	76.09	81.25	4	4
Screwdriver	67.39	50.00	4	3
Corkscrew	63.04	58.33	4	4
Lemon	63.04	72.92	4	4
Dice	60.87	52.08	4	3
Rubber duck	60.87	62.50	4	4
Spoon	58.70	39.58	4	2
Picture frame	56.52	29.17	3	3
Basket	56.52	43.75	3	1
Carrots	56.52	41.67	3	3
Trowel	54.35	43.75	3	2
Book	54.35	31.25	3	3
Tape	50.00	56.25	3	4
Phone	47.83	29.17	3	1
Calculator	47.83	27.08	3	2
Paintbrush	45.65	37.50	3	1
Hat	45.65	25.00	3	2
Pliers	45.65	41.67	3	2
Funnel	43.48	25.00	2	2
Scissors	43.48	58.33	2	2
Rubber spatula	43.48	31.25	2	4
Cork	43.48	54.17	2	4
Camera	43.48	37.50	2	1
Ladle	41.30	37.50	2	2
Plate	39.13	41.67	2	3
Nail file	36.96	41.67	2	2
Pizza cutter	36.96	33.33	2	2
Light bulb	36.96	35.42	2	2
Glasses	34.78	31.25	2	2
Lock	34.78	18.75	2	1
Tennis ball	34.78	47.92	2	3
Hole puncher	32.61	43.75	1	3
Toothbrush	32.61	14.58	1	2
Shell	32.61	31.25	1	1
Mug	30.43	22.92	1	3
Magnifying glass	30.43	43.75	1	1
Oven mitt	30.43	27.08	1	1
Flashlight	28.26	14.58	1	1
Sponge	26.09	29.17	1	1
Paint roller	23.91	20.83	1	1
Grand mean	48.17	42.04	–	–

Percent (%) correct recall and recall quartile are displayed separately for each item and Viewing Condition. Data are ranked according to % correct recall for real object displays.

For each observer, we calculated sensitivity to the study material (d′ in Equation 1), where Hits (H in Equation 1) = the number of items that *were present* in the study set, and which a subject *correctly identified* as being present; False Alarms (F in Equation 1) = the number of items that were *not present* in the study set, but which a subject *incorrectly identified* as being present; and z (in Equation 1) = z-transform. Mean (SD) d′ was calculated across all observers in each Viewing Condition, and compared using ANOVA and follow-up pairwise comparisons where appropriate. Finally, we conducted an Item × Viewing Condition analysis using mixed model ANOVA to examine whether the pattern of recall was similar across items in each viewing condition, with a view to elucidating whether or not the advantage of real object displays on memory performance was related to the types of items we used in our study set.

## Results

### Experiment 1

In Experiment 1 we compared memory performance for stimuli presented in one of three Viewing Conditions: real objects, color photographs, and line-drawings. Recall responses for one subject were absent from the color photograph condition, and so the data were analyzed with 26 subjects in the line drawing and real object groups, and 27 for color photographs. Memory performance in each Viewing Condition was compared using one-way between-subjects Analysis of Variance (ANOVA), followed by planned comparisons between each pair of means (Tukey's honestly significant difference; HSD). The Greenhouse-Geisser correction for violations of sphericity was applied where appropriate for within-subjects analyses.

**Figure 2:**
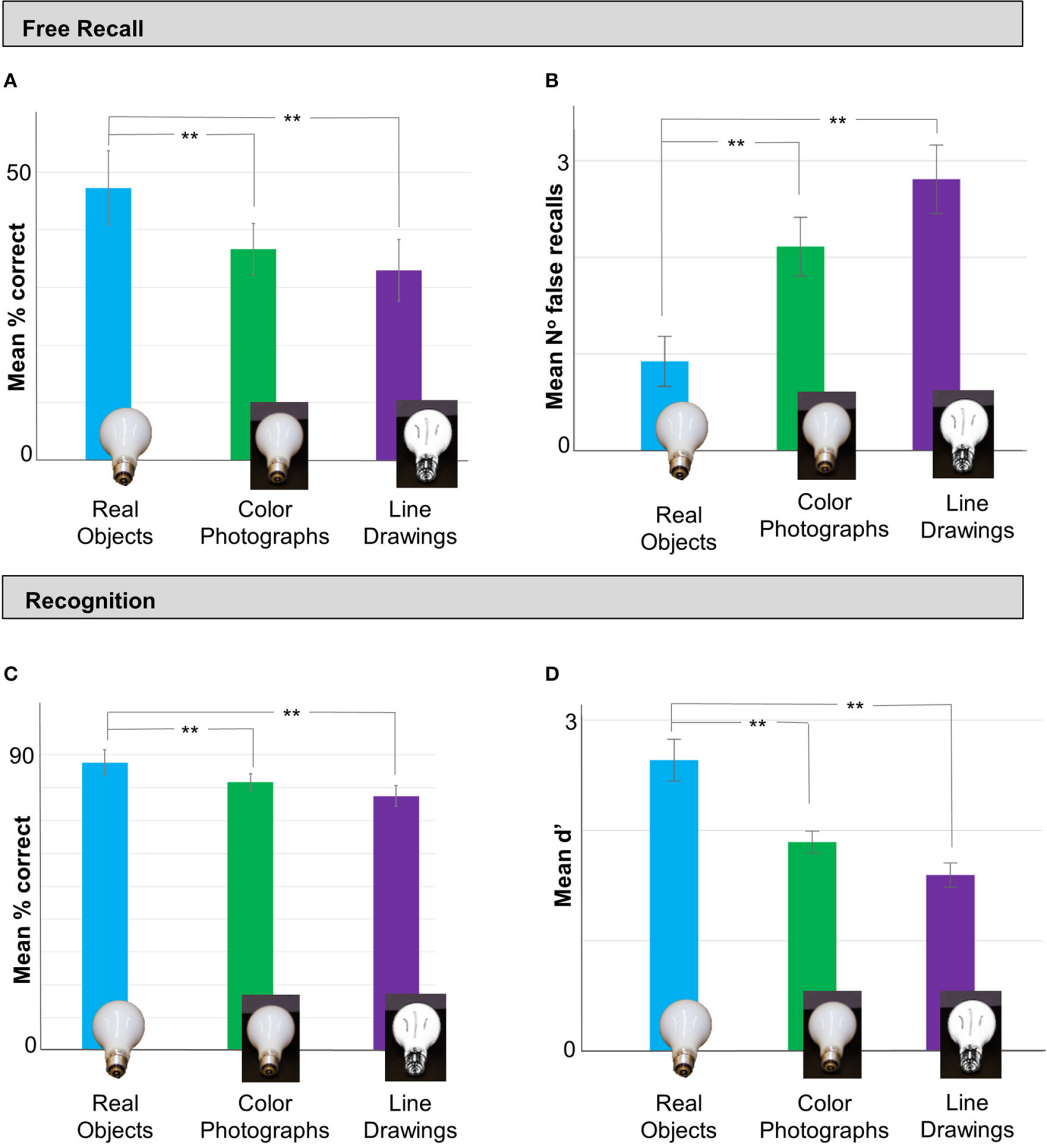
**In Experiment 1 memory performance was better for real objects than line drawings or color photographs**. **(A)** In the Test Phase, free recall (% correct) was better for stimuli in real object displays (blue bar) than as color photos (green bar) or line drawings (purple bar). Importantly, recall was not statistically different in the two image conditions, suggesting that the addition of color and shape cues was not sufficient to enhance memory performance. **(B)** Participants in the real object condition also made significantly fewer false recalls than those in the color photos and line drawing conditions. **(C)** A similar pattern was observed in the subsequent recognition task: recognition (% correct) was significantly better for stimuli shown as real objects than color photos or line drawings, and there was no difference in recognition for stimuli in the color photo vs. line drawing displays. **(D)** Signal detection analyses (mean d′) confirmed that observers who viewed real objects were more sensitive to the study objects than those in the two image conditions. Error bars represent ^**^p < 0.001.

In the free recall task, we observed a significant difference in memory performance for items in each Viewing Condition [*F*_(2, 76)_ = 11.277, *p* < 0.001, η^2^ = 0.229]. Observers' ability to recall real objects (Mean = 49.04%, *SD* = 13.19%) was significantly better than for color photograph (Mean = 36.62%, *SD* = 11.26%) or line-drawing displays (Mean = 34.27%, *SD* = 11.71%); *p* = 0.001 and *p* < 0.001, respectively; Figure 2A). Interestingly, recall performance for color photographs was not significantly different from that of line-drawings (*p* = 0.759), suggesting that additional monocular shape and color cues were not sufficient to facilitate object memory.

Next, we examined the number of *falsely* recalled items—items that participants listed in the recall task as being part of the study set, but were not in fact present (Figure 2B). Interestingly, there was also a significant difference in the number of falsely recalled items across the Viewing Conditions [*F*_(2, 76)_ = 9.42, p < 0.001, η^2^ = 0.199]: participants who viewed real objects made *fewer* false recalls (Mean = 0.93, *SD* = 1.32) than those who viewed color photographs (Mean = 2.11, *SD* = 1.58; p = 0.02, Tukey's HSD) or line drawings (Mean = 2.81, *SD* = 1.81; p < 0.001, Tukey's HSD). There was no difference in mean number of falsely recalled items in the two pictorial conditions (*p* = 0.25, Tukey's HSD). These analyses confirm that observers' superior recall performance of real objects was not simply attributable to producing longer lists of items (thereby inflating the probability of correctly identifying items from the study set due to chance guessing), but demonstrate rather that their knowledge of the study material was also more specific than observers who viewed 2D pictures.

For the recognition task, we also observed a significant difference in % items correctly recalled across Viewing Conditions [*F*_(2,77)_ = 10.359 *p* < 0.001, η^2^ = 0.212; Figure 2C]. Recognition performance was superior for subjects who viewed real objects (Mean = 87.67%, *SD* = 9.04%), vs. colored photograph (Mean = 81.78, *SD* = 6.53; *p* = 0.025, Tukey's HSD) or line-drawing displays (Mean = 77.45, *SD* = 7.74; *p* < 0.001, Tukey's HSD). There was no significant difference in recognition performance for the line-drawing vs. color photograph conditions (*p* = 0.131). To exclude the possibility that differences in recall performance could be explained simply by response biases, we examined the recall data using a Signal Detection (SD) analysis (Figure 2D; see Methods). There was a significant difference in sensitivity to the study material across the different Viewing Conditions [*F*_(2, 77)_ = 14.96, *p* < 0.001, η^2^ = 0.28]. Observers who viewed real objects in the study phase showed significantly greater sensitivity to the study items (Mean *d*' = 2.64, *SD* = 0.97) than those in the color photograph (Mean *d*' = 1.89, *SD* = 0.52; *p* < 0.004, Games-Howell *post-hoc* test for inequality of variance) and line drawing conditions (Mean *d*' = 1.59, *SD* = 0.82, *p* < 0.001, Games-Howell). There was no difference in d′ between the two pictorial conditions (*p* = 0.12, Games-Howell).

Finally, we considered whether there were commonalities in the *types* of stimulus items that were recalled in the real object vs. pictorial viewing conditions. Our stimulus set was varied and the items may be categorized a number of ways (i.e., fruits vs. non-fruits, natural vs. man-made objects, tools vs. non-tools, etc.). We examined observers' recall performance across Items in each Viewing Condition using a mixed-model 2-way ANOVA with the between-subjects factor of Viewing Condition (real, photographs, line drawings) and the within-subjects factor of Item ID (*n* = 44). This analysis revealed a main effect of Viewing Condition [*F*_(2, 76)_ = 11.28, *p* < 0.001, η^2^ = 0.23], and Item [*F*_(25.16, 3268)_ = 7.50, *p* < 0.001, η^2^ = 0.09], and a significant Viewing Condition × Item interaction [*F*_(50.33, 3268)_, *p* < 0.001, η^2^ = 0.05], suggesting that recall performance differed across items as a function of Viewing Condition. Table 1 presents percent recall data for each item in each viewing condition, and the quartile into which each item fell in % recall (e.g., with items in the 4th quartile being recalled more frequently than those in the 1st). Items that were recalled *most* frequently in the real object displays (e.g., 4th quartile: flashlight, soap, lemon, dice) but less so in the pictorial conditions (below 4th quartile) fell into a range of categories including man-made and natural objects, tools and non-tool objects, and objects without scent (note that all fruit/vegetable items were made of plastic). Taken together, the recall and recognition data from Experiment 1 indicate that memory is enhanced for real object displays, and that this enhancement generalizes across a range of stimulus sub-categories.

It is possible, however, that the improved memory performance for stimuli displayed as real objects could be attributed to differences in viewing angle of the stimuli across the real vs. pictorial conditions. Although the 2D stimuli were generated by photographing the real objects mounted in the presentation box (thereby allowing control of stimulus position, order, and orientation across conditions), the 2D images were *themselves* mounted at an angle of 30° on the display shelves, which further increased viewing angle relative to the real objects—possibly making the 2D images more difficult to identify. Although none of our observers complained of an inability to recognize the stimuli (and outlier stimuli were filtered in the initial item analysis described above), it is nevertheless possible that a subtle increase in difficulty in stimulus identification could have manifested as poorer recall/recognition performance. We examined this possibility in a follow-up experiment.

### Experiment 2

In Experiment 2 we compared memory performance for real objects vs. color photographs in a separate group of observers. Importantly, in Experiment 2 the real object stimuli were presented (and photographed to create matching 2D images) on “shelves” that were oriented vertically, rather than at 30° as in Experiment 1, to match viewing angle across the real object and photograph conditions (Figure 1B). Free recall and recognition performance were compared using planned comparisons between the Viewing Conditions (two-tailed independent-samples *t*-tests, significant at *p* < 0.05).

**Figure 3:**
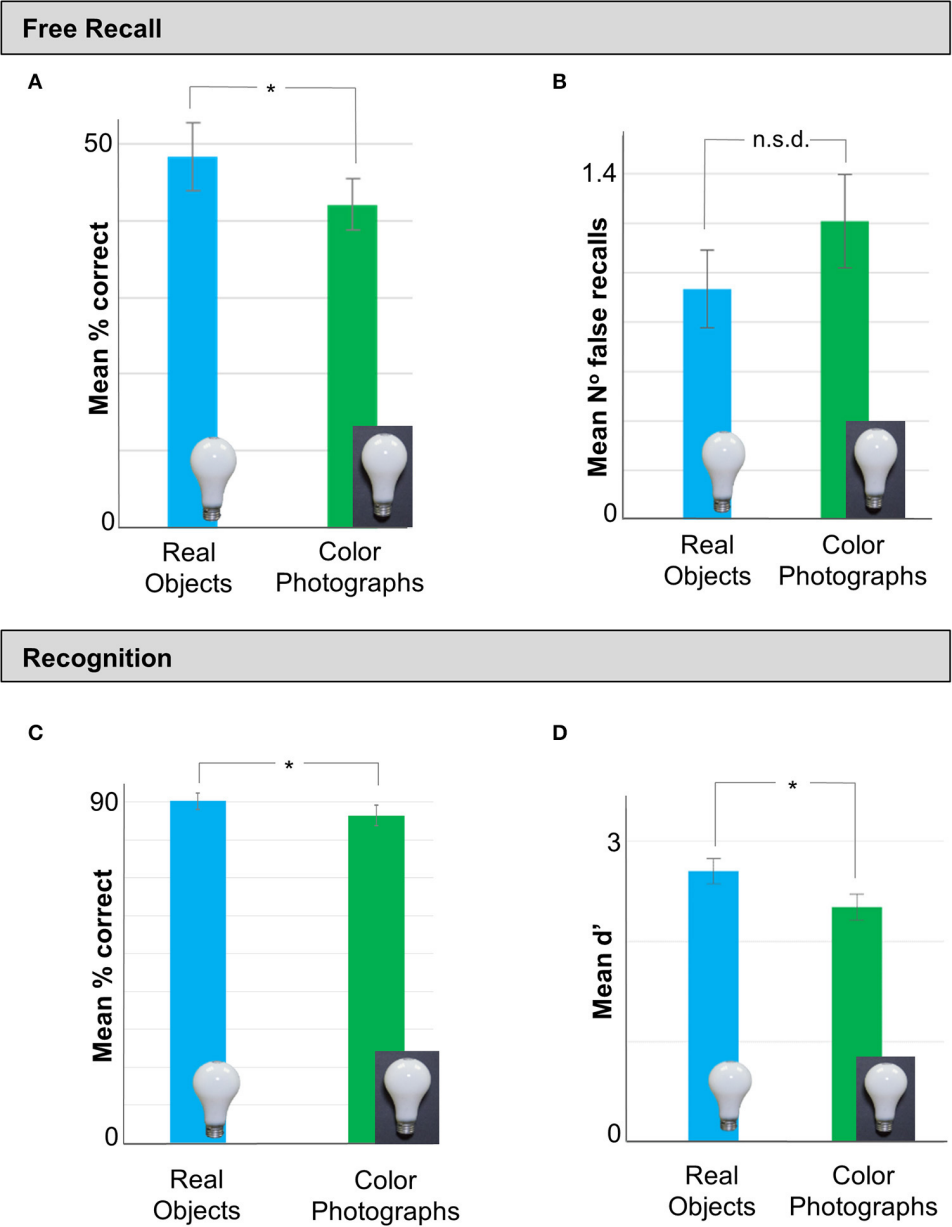
**Data from Experiment 2 in which we tested memory performance for real objects vs. color photographs in a separate set of observers. (A)** As in Experiment 1, stimuli presented as real objects in the Study phase were recalled significantly better than color photograph displays of the same items. **(B)** There was no difference in the number of falsely recalled items between real objects and photos. **(C)** Analysis of the recognition data revealed that participants in the real object condition made an equivalent number of false recalls as observers in the color photos condition. **(D)** Finally, a SD analysis of the recall data revealed that observers who viewed real objects were significantly more sensitive to the study material than those who studied color photos of the same objects. Error bars represent *SE*. ^*^p < 0.05.

As in Experiment 1, observers recalled a greater number of items in the real object (Mean = 48.17%, *SD* = 14.86%) than the color photograph condition [Mean = 42.05%, *SD* = 11.48%; *t*_(90)_ = −2.149, *p* = 0.034, Cohen's *d* = −0.45] (Figure 3A). Although there were again fewer falsely recalled items in the real (Mean = 0.93, *SD* = 1.05) than the color photograph (Mean = 1.21, *SD* = 1.30) condition (Figure 3B), this difference did not reach statistical significance [*t*_(90)_ = 1.11, *p* = 0.27]. Recognition performance (% correct) was also significantly better for real objects (*M* = 90.24%, *SD* = 7.38%) than color photographs [Mean = 90.24%, *SD* = 7.37%; *t*_(90)_ = −2.261, *p* = 0.026, Cohen's *d* = −0.47] (Figure 3C). A SD analysis of the recognition data revealed that sensitivity (mean d′) was significantly higher for real objects (Mean = 2.70, *SD* = 0.86) than colored photographs (Mean = 2.33, *SD* = 0.91) (Figure 3D), [*t*_(90)_ = 2.03, *p* = 0.045, Cohen's *d* = 0.43].

In summary, Experiment 2 replicated the findings of Experiment 1 showing that free recall and recognition performance for real objects was significantly better than matched color photographs of the same items. Importantly, Experiment 2 confirmed that the memory advantage for real objects observed was not attributable to subtle differences in viewing angle of the items across conditions.

Finally, as in Experiment 1, we examined observers' recall performance across Items in each Viewing Condition using a mixed-model 2-way ANOVA with the between-subjects factor of Viewing Condition (real, photographs) and the within-subjects factor of Item ID (*n* = 44). We observed a main effect of Viewing Condition [*F*_(1, 92)_ = 5.03, *p* < 0.001, η^2^ = 0.05], and Item [*F*_(25.635, 3956)_ = 6.00, *p* < 0.001, η^2^ = 0.06], but no higher-level interaction effect [*F*_(50.33, 3268)_ = 1.14, *p* = 0.29, η^2^ = 0.01], indicating that although some items were recalled more frequently than others, the pattern of recall performance for the various items was similar across the different viewing conditions. Table 2 presents percent recall data for each item in each viewing condition, and the quartile into which each item fell in percent recall in each Viewing Condition. As in Experiment 1, items that were recalled more frequently from real object (3 rd–4th quartiles) than color photograph displays fell into a range of different sub-categories.

## Discussion

We compared episodic memory for everyday household objects that were viewed as real 3D objects vs. 2D pictures. In Experiment 1 free recall and recognition performance were examined for identical stimulus sets that were viewed in one of three different display formats: real objects, color photographs, and black and white line drawings. Memory performance was superior for stimuli that were displayed as real 3D objects in the Study Phase than line drawings or color photographs of the same stimuli. Analysis of erroneous responses (falsely recalled items, and signal detection analysis of the recognition data) confirmed that observers who viewed real objects were indeed more sensitive to the study material than those who viewed the same stimuli in pictorial form. In Experiment 2, free recall and recognition performance were compared for real objects vs. color photographs when viewing angle was matched across the different display conditions. Again we found superior memory performance for real objects over color photo displays, and the pattern of data could not be explained by effects of response bias. Finally, item-based analyses of the recall data in Experiments 1 and 2 indicated that the memory advantage for real objects generalized across a range of different stimulus types, including man-made and natural, tools and non-tool objects. Taken together, our data demonstrate for the first time that real objects are more memorable than picture representations.

We argue that the influence of real object displays on memory is due to a fundamental mnemonic advantage for real objects. In terms of alternative interpretations we considered the possibility that there was a systematic difference in encoding strategy adopted by observers in the different viewing conditions. This account seems unlikely for several reasons. The stimuli in each condition were presented in the same order and position on the display shelves, such that the spatial and semantic relationships between the items were constant across conditions. Viewing time was also equivalent across conditions. Further, given our large sample size it is unlikely that all subjects within a given group employed the same encoding strategy and that these strategies were reliably different across groups but consistent across experiments.

Below, we discuss several factors that might be important in driving the real object advantage in memory. As outlined in the introduction, binocular vision provides information about the geometric structure of real objects that is not available when looking at 2D images (Blake and Wilson, 2011). The memory advantage for real objects over images may be due to additional binocular cues to 3D depth structure in our real object displays. If additional shape cues facilitate memory, the question arises as to exactly what *type* of shape cues? In Experiment 1, memory performance was equivalent in the black and white line drawing and color photo condition, suggesting that color and monocular shape cues (i.e., shading and surface texture) were insufficient to influence object memory, despite the fact that color and other surface cues have been shown to have influence on object identification (Humphrey et al., 1994). The possibility remains however, that additional binocular stereo cues to shape could enhance memory performance. Although depth information from disparity may have a modest effect on the time taken to *recognize* an object (Edelman and Bulthoff, 1992; Humphrey and Khan, 1992), this result has not been supported in all studies (D'erme et al., 1994). Interestingly, previous studies comparing memory and other cognitive measures for 2D displays with matched virtual 3D computer-generated displays have found that performance actually declines when observers move from 2D to 3D conditions—at least in cluttered environments such as digital aviation panels, or navigating complex web-pages (Wickens et al., 1996; Risden et al., 2000; Cockburn and Mckenzie, 2002). Valsecchi and Gegenfurtner (2012) reported a stereo-viewing enhancement in long term memory for forest pictures, even when subjects had no explicit memory of the format in which the stimuli had originally been displayed. Interestingly, this memory enhancement was contingent on lengthy (7 s) display times and was not apparent across all stimulus categories: stereo viewing did not enhance recognition of other scene categories such as car and house images. Valsecchi and Gegenfurtner (2012) concluded that the beneficial effect of stereo information on memory is apparent only when the 3D structure of the object or scene is relevant to the subject's task (i.e., spatial layout), and when observers have sufficient time to study the image. With simple displays containing isolated objects, such as those used here, memory may be less influenced by task-irrelevant or otherwise distracting visual information, thereby revealing a beneficial effect of 3D stereo cues on memory relative to 2D images. Critically, however, none of these studies of stereo vision have compared memory for virtual 3D object displays with *real* 3D objects.

It is interesting to consider whether the real object advantage might be explained by other higher-level attributes intrinsic to real objects. Real objects are tangible substances that exist in 3D space, with a definite surface texture, compliance, and function. Images, conversely, are abstract representations of objects that must be learned during childhood to be fully understood (Deloache et al., 2003). Perhaps most importantly, objects that are placed within reaching distance afford action, such as grasping and manipulation (Gibson, 1979; Norman, 2002) whereas images of objects do not. Indeed, graspable objects are particularly relevant for dorsal-stream motor networks (Chao and Martin, 2000; Creem-Regehr and Lee, 2005; Handy et al., 2006; Proverbio et al., 2011). Several of these areas are known to be highly sensitive to whether a real graspable object is within or outside of reach (Gallivan et al., 2011). The objects in our study were presented within reach of all subjects, thereby reasonably offering affordances. Dorsal stream regions within inferior parietal cortex are known to be active during working memory (Todd and Marois, 2004; Xu and Chun, 2006), episodic memory (reviewed in Wagner et al., 2005; Cabeza et al., 2008; Vilberg and Rugg, 2008), and motor planning (Chao and Martin, 2000; Handy et al., 2003; Creem-Regehr and Lee, 2005; Handy et al., 2006; Proverbio et al., 2011; Makris et al., 2013; Garrido-Vasquez and Schubo, 2014). Our results raise the intriguing possibility that real objects may be more memorable because they more strongly activate dorsal stream regions at encoding, perhaps promoting deeper processing and superior memory. In other words, real objects may have a memory benefit due to embodied cognition (Glenberg, 1997; Barsalou, 2008). There are some data to support this interpretation, showing differential neural responses when maintaining objects with and without affordances in memory. For example, Mecklinger et al. (1998) examined the role of motor affordances on working memory using object images. These authors found that maintaining manipulable objects in memory increased ventral premotor cortex activity whereas maintaining non-manipulable objects in memory activated inferior frontal gyrus. In line with this idea, previous studies have reported memory improvements for images of graspable objects (Apel et al., 2012; Downing-Doucet and Guerard, 2014); but see, Pecher (2013) and Quak et al. (2014). It should be noted however, that thus far, *all* of these studies used 2D pictures of objects (which do not afford action in and of themselves), rather than real world exemplars.

It is possible that the real objects in our study were perceived as being more useful or valuable (perhaps because of their direct relevance for action), thereby influencing how memorable they were. In a recent study, Bushong et al. (2010) gave college students money to “bid” on different types of snack foods, which (depending on the bid) could be purchased at the end of the study. Using a between-subjects design, the students viewed the snack foods in the bidding phase in one of three different viewing conditions: real foods, color photographs of the same foods, or a text display of the snack food name. Bushong et al. (2010) found that students were willing to pay over 60% more for foods that were displayed as real objects vs. image or text displays. The same effect was replicated using small trinkets. In the domain of human memory, previous studies have shown that items associated with a higher incentive can be remembered strategically better than items with a lower perceived payoff (Castel et al., 2002). To the extent that our real everyday objects were perceived as being more valuable than the matched image displays, this could also have resulted in a beneficial mnemonic influence.

Real objects have an unambiguous size, distance, and location relative to the observer. In the current study stimuli in all viewing conditions were matched for retinal size. It is the case, however, that observers in the picture conditions were not explicitly aware that the size of the images corresponded to the real world size of the objects. Recent evidence suggests that information about the real world or “familiar” size of objects is accessed relatively automatically (Konkle and Oliva, 2012a), and may be a guiding principle in the large-scale organization of object representations in occipitotemporal cortex (Konkle and Oliva, 2012b). It is interesting to speculate as to whether the real object benefit is heightened in our study by permitting immediate access to stored representations of object identity in object-selective cortex, relative to those who viewed images and whose size information is not explicit. Complementing these findings, damage to inferior occipitoparietal cortex can disrupt distance perception and motor planning that can be partially rescued by object familiarity (Berryhill and Olson, 2009).

In conclusion we found that memory for real objects was significantly better than 2D image representations of the same exemplars. Our data shed important new light on the fundamental yet largely overlooked question of whether pictures are an appropriate proxy for real objects in psychology and neuroscience. These results pave the way for more detailed investigations of the mechanism for the memory advantage for real objects and their underlying neural basis. The findings reported here suggest that although convenient, the use of images in memory research is likely to underestimate memory performance and to reflect incomplete mnemonic processing.

### Conflict of interest statement

The authors declare that the research was conducted in the absence of any commercial or financial relationships that could be construed as a potential conflict of interest.
